# Exploration of the administrative aspects of the delivery of home health care services: a qualitative study

**DOI:** 10.1186/s12930-018-0038-x

**Published:** 2018-01-24

**Authors:** Hooman Shahsavari, Alireza Nikbakht Nasrabadi, Mohammad Almasian, Heshmatolah Heydari, Abdolrahim Hazini

**Affiliations:** 1 Department of Medical-Surgical Nursing, School of Nursing and Midwifery, Tehran University of Medical Sciences, Tehran, Iran; 2 School of Medicine, Lorestan University of Medical Sciences, Khorramabad, Iran; 3 Social Determinants of Health Research Center, Lorestan University of Medical Science, Khorramabad, Iran; 4 Department of Community Health Nursing, School of Nursing and Midwifery, Lorestan University of Medical Sciences, Khorramabad, Iran; 5 Department of Home-based Palliative Care, ALA cancer prevention and control of charity center (MACSA), Tehran, Iran

**Keywords:** Home health care, Home care agencies, Nursing, Administration, Qualitative study, Iran

## Abstract

**Background:**

Because of the variety of services and resources offered in the delivery of home health care, its management is a challenging and difficult task.

**Objectives:**

The purpose of this study was to explore the administrative aspects of the delivery of home health care services.

**Methods:**

This qualitative study was conducted based on the traditional content analysis approach in 2015 in Iran. The participants were selected using the purposeful sampling method and data were collected through in-depth semi-structured personal interviews and from discussions in a focus group. The collected data were analyzed using the Lundman and Graneheim method.

**Results:**

23 individuals participated in individual interviews, and the collected data were categorized into the two main themes of policymaking and infrastructures, each of which consisted of some subcategories.

**Conclusion:**

Health policymakers could utilize the results of this study as baseline information in making decisions about the delivery of home health care services, taking into account the contextual dimensions of home care services, leading to improvements in home health care services.

## Background

As life expectancy rises and chronic conditions become more common and the population ages, the use of nursing services at home is an inevitable and undeniable phenomenon [1]. Home care includes nursing, medical, rehabilitative, and social services [2]. These services include a wide range of primary and advanced services offered at three levels of prevention by different individuals from health care professionals to volunteers, such as friends, family members, and neighbors [2]. This type of care leads to shorter hospital stays, fewer readmissions, fewer and less serious hospital-acquired complications, a reduced need for repeated referrals to the emergency departments, the shortening of the waiting time in treatment centers, vacancy of specialty hospital beds, decreased emergency department crowding, improving the quality of life and savings in health care expenses [3–5]. The management of the delivery of home care services is a challenging and difficult task, because of the variety of services needed, the variety of resources needed to offer the services, and shortage of human resources [6].

The management and provision of home care services in each society depends on the cultural context of the society, the available infrastructures, and the structure of the health system of that country [7]. As a developing country in the Middle East, Iran has to deal with various ethnic and cultural groups, and the phenomenon of an aging population, and, as a result, the heavy burden of chronic diseases [8]. In this country, various levels of health services are offered to the society by universities of medical sciences in each province [9].

In Iran, nurses will be licensed to establish nursing counseling and nursing services institutes by the Ministry of Health and Medical Education. In these institutions, different groups of medical professionals can serve at different levels of primary, secondary and tertiary prevention and care alongside each other, based on the needs of the patients and their families. The technical team includes general practitioners and specialist doctors, and the nursing team, includes nurses at different levels of education (B.Sc., M.Sc., and Ph.D.), operating room and anesthesiology technicians, medical emergency technicians, rehabilitation technicians, and psychologists at different levels of education, with valid certificate provide specialized services based on their fields in home nursing care centers. Non-technical members of the centers include nurse-aids and practical nurses or nurse assistants who have received a valid certificate of basic services from non-university centers and have the competence to provide basic services to clients [10]. Managing and coordinating the process of providing services to clients via home care centers is the responsibility of qualified nurses. Nurses are not licensed to prescribe medications in the Iranian health system [11]. Additionally, terminally ill patients rarely receive their health services from home care institutes, and most of them are hospitalized during the last days of their lives and pass away in hospitals [5]. Managing the provision of home care services is difficult and challenging due to the diversity of services offered, the variety of resources required to provide services, and labor shortage. The diversity of patient needs, the qualifications of caregivers, the high workload of caregivers, the diverse demands made on the organization, the long distances that the caregivers have to travel to the residences of the patients are among the factors that make the provision of home nursing services challenging [12, 13]. According to some studies, home care services in Iran face a number of challenges, including deficiencies in policymaking, lack of resources, incomplete guidelines, the fact that there is inadequate familiarity with this type of care in the society, and the lack of adequate regulations and directives on how to provide home care services [13, 14].

Naturally, proper planning is required for home care, and before planning, it is necessary to investigate the target phenomenon from different management perspectives, so that its various dimensions are well understood, so that the stakeholders can have a deeper understanding of the target phenomenon in order to make the right decisions. Therefore, given the absence of a study in this regard and the long-term experiences of the authors in providing home care services, it seemed that conducting a qualitative study could help in achieving a deep understanding of this phenomenon.

### Objectives

This study aimed to explore the administrative aspects of the delivery of home health care services.

## Methods

### Design

This qualitative study, which had a content analysis approach, was conducted in 2015 in Iran. It was attempted to achieve maximum variability and enroll information-rich participants in order to significantly facilitate an in-depth study and to advance toward the core objectives. Therefore, nurses, physicians, caregivers, policymakers, patients and their families that were familiar and had experience with home health care were selected using the purposeful sampling method. The inclusion criteria were: having adequate experience in the provision of home health care, being communicative and expressive, and being willing to participate in the study.

### Data collection

Data were collected through in-depth semi-structured individual interviews and from discussions in a focus group. All the interviews were conducted in a quiet and private place and were recorded using a digital voice recorder. The guiding questions of the interview were as follows: “Can you talk about home health care? What do you think about home nursing care? How should home health care be directed?” During the interview, exploratory and probing questions were asked from the interviewees to clarify the issues. At the initial stages of the study and during interviews, the research questions were semi-structured, but over time, the questions became more structured. Sampling and interviews continued until saturation was achieved [15]. The duration of each face-to-face interview was between 20 and 40 min. In order to obtain richer data and obtain data from group discussions, a session of the focus group was held.

### Data analysis

Based on the views of Lundman and Graneheim, data analysis was conducted simultaneously with the interviews [15], such that immediately after each interview, the statements were written down by hand and typed verbatim. Next, the handwritten texts were studied several times and the initial codes were extracted. After that, related codes were merged based on their similarities and differences, categories and themes were formed, and finally the concepts that were hidden in the data were extracted.

### Trustworthiness

Based on the views of Lincoln and Guba, in order to ensure the accuracy and reliability of the data, the criteria of credibility, confirmability, and trustworthiness were used [16]. For this purpose, the researcher had long-term contact with the participants. After the initial analysis, the codes were discussed with the participants to ensure their accuracy, and if the codes were not consistent with the views of the participants, corrections were made, so that the researcher and the participants reached agreements on the codes. Furthermore, discussions were held among the members of the research team to reach agreements on the selected codes and the categories and themes that had emerged. The participants in the study greatly varied in terms of age, gender, educational attainment levels, work experience, length of their work experience, and the type and location of their occupations during their careers.

### Ethical considerations

This study was examined by the ethics committee of the Tehran University of Medical Sciences and received a written permission with the code of IR.TUMS.REC.1394.175. Written informed consents were obtained from the participants.

## Results

23 individuals were interviewed in person, including 11 men and 12 women. One of the participants was interviewed three times and another participant was interviewed twice. Overall, 25 individual interviews were performed. The participants in the focus group were 7 individuals including 5 men and 2 women.

After data analysis, data were placed in the two main themes of policymaking and infrastructures, each of which consisted of some subcategories (Table 1).

**Table 1:** The classes and subclasses of the dimensions of home care

Main categories	Subcategories
Policymaking	Using multi-dimensional management in the structure of the health system
	Nurse as the axis of the home care team
	Community orientation in health system
Infrastructure	Readiness of the Social context
	Societal understanding of home care
	Societal understanding of nurses
	The views of professionals regarding home care
	The coverage of the services by universal insurance
	Suitable electronic communications
	Adequate knowledge of home care
	The training of professionals
	The training of the patients’ families
	Inter-professional cooperation
	Inter-sectoral cooperation

### Policymaking

The analysis of the data showed that methods of policymaking could affect the offering of health services to the clients. This theme itself consists of the three categories of using multidimensional management in the structure of the health system, taking account of the role of nurses as the axis of the home care team, the necessity of community orientation in the health system.

#### Using multidimensional management in the structure of the health system

The analysis of the data indicated that the participants mentioned that physician-centrism is a barrier to the development of home nursing care. In this regard, a nurse practitioner active in the policymaking field stated that

“… There are negative views regarding home care, and the reason is that the medical system is physician-centrist…. They think their wallets would suffer….” (Participant number 5).

#### Nurses as the axis of home nursing care

The analysis of the data indicated that one of the most important members of the home care team is the nurse. In this regard, a specialist physician stated that

“… the nurse knows more about the patient’s problems than other members of the health care team, because he/she spends more time with the patient, and it is the nurse that can act as the guide for the health care team….” (Participant 5).

#### Community orientation in the health system

Data analysis showed that, to enrich home nursing care, it is necessary that the health system does not limit health services to the hospital.

In this regard, one of the instructors emphasized the importance of the provision of health services at the level of the community, and stated that

“…If the intention of the health system is to break this cocoon, sitting at health and treatment centers and expecting to be able to do something is useless, …” (Participant 4).

### Infrastructures

In order to reach the goals, some factors play a fundamental and basic role, and the activities of the other parts of the system depend on the correct functioning of these factors. This theme consists of six categories including, readiness in the social context, the coverage of the services by universal insurance, suitable electronic communication infrastructures, adequate knowledge, inter-professional and inter-sectoral cooperation for the provision of home nursing care.

#### The readiness of the social context

Data analysis suggested that social class, economic and cultural status, beliefs, norms, and the understanding of members of the society regarding home nursing care services can directly influence the extent to which families take advantage of home care, and the way these services are offered, and can facilitate or hinder the progress of home nursing care programs.

##### Social perception of home care

Data analysis showed that society’s understanding and interpretation of home nursing care could affect the modes of utilization of home care. In this regard, a physician in charge of an institute offering home nursing care stated that

“… ultimately you may culturally reach conclude that if you receive such a service at home, it is less costly for you, less troublesome, and more convenient, and your dignity and respect is preserved…” (Participant 9).

##### Society’s perception of the nurses

The participants in this study believed that, for the provision of home care to flourish, it is necessary that members of society have a positive view towards the nurse, and consider him/her as an educated, qualified, reliable person who is effective in the patient’s recovery process. In this regard, one of the participants stated that

“…. People think that nurses can only perform injections and infusions… Unless we represent the nurse as a professional… a medical professional, who is as skillful as a G.P… home care will not be successful …” (Participant 2).

##### The viewpoints of practitioners towards home care

The participants in this study believed that the interpretation of specialists regarding the benefits of home care, the extent to which they trust the process of the provision of home nursing care services, and their cooperation in recommending home nursing care to patients and their families could play an important role in the success of home care programs.

In this regard, a professional active in the field of home care states

“… doctors prefer patients to remain in the hospital because of the financial benefits ….” (Participant 8).

#### Coverage of home care services by universal insurance

Data analysis indicated that home care involves high costs and the coverage of home care by universal insurance is beneficial. In this regard, one of the participants talks about the benefits of home care services being covered by insurance companies:

“… If insurance companies cover a percentage of the costs, people won’t turn to illegal centers … and [these services] become regulated…” (Participant 5).

“…the coverage of home care services by universal insurance has been emphasized by the WHO…” (The Focus Group).

#### Suitable electronic communications

Data analysis suggested that if good communication systems are available, home care providers (nurses, caregivers, etc.) can easily keep in touch with the other members of the care team, and provide the services needed by the patient quickly.

#### Adequate knowledge of home care

Data analysis showed that a factor that can affect the provision of home nursing care is academic training in universities and the training of families when they need home nursing care.

##### The training of professionals

Data analysis emphasized community-oriented training of the nurses, which can be included in the educational curriculums. In this relation, a specialist physician says that home care nurses should be better versed and more skillful in the services they provide than hospital nurses and states

“… All training programs for nurses should be changed.… Since the equipment and the setting are no longer clinical, and the nurse must provide a totally different type of service to society …” (Participant 2).

##### Training the patient’s family

The participants believed that, before starting the home nursing care services, it is necessary that the patients and their families receive general training from the center providing home care services, so that family members will be able to participate in the care of the patient and as a result, the costs can be reduced.

#### Inter-occupational cooperation

Data analysis showed that home care involves teamwork, and it is necessary that different health professionals cooperate to provide services to the patient. A nurse stated

“… No one can be an expert in all fields.… For example, a patient might develop bedsores.… I may not have adequate information and knowledge about bed sores, then I should consult my colleagues.…” (Participant 7).

#### Inter-sectoral cooperation

Data analysis showed that it is essential that various organizations should contribute to health care efforts. One of the participants said:

“… Municipalities should cover home care services. Health services belong to municipalities…” (Participant 2).

#### The management of the provision of home nursing care

Data analysis suggested that setting and making use of proper criteria for the recruitment of personnel, the management of financial resources, and the care process are factors that can contribute to the success of home nursing care.

##### Factors related to human resources

Data analysis indicated that it is necessary that home care institutes take great care in the recruitment and hiring of the needed work force, so that they can hire qualified and experienced individuals that can provide appropriate and satisfactory services to the patients. In this regard, the manager of a home care center says

“… Our nurses should be able to perform catheterization, gavage, nasogastric intubation, ECG, injections, and venipuncture.…” (Participant 9).

##### Financial resources

Data analysis showed that financial turnover is a vital component of every organization, such that proper and regular auditing can help the progress of the organization, and leads to the satisfaction of the employees and customers. In this regard, a nurse active in policymaking said about the importance of tariff regulations:

“… They can classify it. … An elderly patient who has Alzheimer’s must pay more than a patient who is alert and not an invalid. … Based on this, they can give us a range, for example from three hundred to four hundred and fifty dollars…” (Participant 6).

A nurse offering home care services said,

“… Minimum and maximum rates should be set…” (Participant 7).

A nurse active in policymaking said,

“…Some individuals work on a part-time basis and are not full-time employees. … They are in fact contractors …. A large part of the tariffs belongs to them. …” (Participant 16).

#### The process of the provision of home nursing care services

Data analysis showed that, in the provision of home nursing care, it is essential that the admission process, the provision of care services, and the evaluation of the provided services should be done based on specific and predetermined plans and programs.

“… After taking the history of the patient and considering all the obtained information, a specific protocol is prepared for the patient….” (Participant 14).

## Discussion

In this study, data analysis revealed that policymaking and infrastructures could be two vital factors in the management of the delivery of home health care services.

In the policymaking dimension, community orientation of the health system is among the factors that can affect home care [17]. Therefore, it is necessary that policymakers propel the health system towards community-centered services. Another factor in the policymaking dimension that can help with the advancement and promotion of home care programs is the use of multi-dimensional management in the administration of the health system, such that the health system takes advantage of the capacities of competent experts at various management levels. In this regard, the World Health Organization has mentioned that physician-centrism in the management of health systems is a grave challenge [18]. According to the WHO, if the health systems want to be able to adequately respond to the health needs of the society, they must be aware of the role that nurses and midwives play in these systems [18].

A key member of the home care team is the nurse [19]. Therefore, it is necessary that the needed arrangements be made for the recruitment and employment of the needed work force.

One of the factors that can play an important role in the provision of home care services is the coverage of home care services by universal health insurance. WHO has emphasized the by universal insurance coverage and the reduction of the payments that people make to receive health services, which is a movement that is represented by the expression Universal Health Coverage (UHC), according to WHO [20]. In this regard, lack of insurance coverage and traditional management causes people to receive these services from unlicensed institutes and caregivers [21].

Educating and training health, nursing, and medicine graduates in providing services outside treatment centers is a vital dimension [22]. Therefore, educational institutes should include courses on home care for various groups of health graduates, especially nurses, to prepare them for working in communities.

One infrastructure that is necessary for the provision of home care services is inter-sectoral and inter-occupational cooperation. The provision of home care services involves teamwork [23], on the other hand, cooperation and coordination between hospitals and home care teams are undeniably crucial, and can help reduce re-admissions and improve service quality.

Another important infrastructure is the existence of executive and administrative protocols for the provision of home care services, which can help with the organization of home care services, the recruitment of human resources, the estimation of the fees and the costs, and the design of the care process [24]. These protocols can help with the assessment of the needs of the clients and their economic and financial status [25]. Therefore, given its own economic, social, and cultural condition and its health system, each country should compile standard instructions for the provision of home care services.

## Limitations

This was a qualitative study conducted on a limited population and generalizations from this study may not be easily feasible.

## Conclusion

It is necessary to transform the delivery of home health services from treatment-centrism to community orientation. In addition, taking account of contextual dimensions, such as the insurance coverage of home care services, the use of pre-determined protocols, improvements in inter-occupational cooperation, and the use of the capacities of other organizations are vital elements for the management of the delivery of home health care services.
